# DNA Barcoding of Birds at a Migratory Hotspot in Eastern Turkey Highlights Continental Phylogeographic Relationships

**DOI:** 10.1371/journal.pone.0154454

**Published:** 2016-06-15

**Authors:** Raşit Bilgin, Nadin Ebeoğlu, Sedat İnak, Mehmet Ali Kırpık, Joshua J. Horns, Çağan H. Şekercioğlu

**Affiliations:** 1 Institute of Environmental Sciences, Boğaziçi University, Bebek, 34342, Istanbul, Turkey; 2 Kafkas University, Faculty of Science, Department of Biology, TR-36100 Kars, Turkey; 3 University of Utah, Department of Biology 257 S 1400 E, Salt Lake City, UT 84112, United States of America; 4 College of Sciences, Koç University, Rumelifeneri, Istanbul 34450, Turkey; 5 KuzeyDoğa Derneği, Ortakapı Mah. Șehit Yusuf Cad., No: 93/1, 36100 Kars, Turkey; University of Veterinary Medicine Hanover, GERMANY

## Abstract

The combination of habitat loss, climate change, direct persecution, introduced species and other components of the global environmental crisis has resulted in a rapid loss of biodiversity, including species, population and genetic diversity. Birds, which inhabit a wide spectrum of different habitat types, are particularly sensitive to and indicative of environmental changes. The Caucasus endemic bird area, part of which covers northeastern Turkey, is one of the world’s key regions harboring a unique bird community threatened with habitat loss. More than 75% of all bird species native to Turkey have been recorded in this region, in particular along the Kars-Iğdır migratory corridor, stopover, wintering and breeding sites along the Aras River, whose wetlands harbor at least 264 bird species. In this study, DNA barcoding technique was used for evaluating the genetic diversity of land bird species of Aras River Bird Paradise at the confluence of Aras River and Iğdır Plains key biodiversity areas. Seventy three COI sequences from 33 common species and 26 different genera were newly generated and used along with 301 sequences that were retrieved from the Barcoding of Life Database (BOLD). Using the sequences obtained in this study, we made global phylogeographic comparisons to define four categories of species, based on barcoding suitability, intraspecific divergence and taxonomy. Our findings indicate that the landbird community of northeastern Turkey has a genetical signature mostly typical of northern Palearctic bird communities while harboring some unique variations. The study also provides a good example of how DNA barcoding can build upon its primary mission of species identification and use available data to integrate genetic variation investigated at the local scale into a global framework. However, the rich bird community of the Aras River wetlands is highly threatened with the imminent construction of the Tuzluca Dam by the government.

## Introduction

Birds are found in all habitat types and are sensitive to environmental changes. Therefore, they are good indicator species for environmental monitoring [1]. Main causes of bird declines include habitat loss, exploitation (e.g. hunting), introduced species, and pollution [2], with climate change rapidly becoming a major threat [1, 3]. Increasing temperatures also force many species to move to higher altitudes, reducing their ranges, particularly threatening tropical montane endemics and species of extensive lowlands [3, 4].

DNA sequences are significant sources of information for improving our understanding of the evolutionary and genetic relationships of organisms [5]. In the last decade, DNA barcoding has become an increasingly important tool to catalog the diversity of life and to improve taxonomic classification. DNA barcoding is based on the notion that a short standardized sequence can differentiate the individuals of a species from those of other species because the sequence variation between species is assumed to be more than that within. The main objectives of DNA barcoding are to help the identification of unknown specimens, speed up the designation of distinct lineages, and improve the discovery of new species. Through DNA barcoding, a number of cryptic species, which were previously thought to be a single species, have also been discovered [6]. On the other hand, there are some controversies about using single gene thresholds to discover new species especially considering recent divergences [7].

Mitochondrial gene cytochrome c oxidase I (COI, cox1) is frequently used for DNA barcoding in animals. Studies show that more than 95% of species possess unique COI barcode sequences, so species-level identifications can generally be done successfully [6, 8]. In essence, DNA barcoding helps in the characterization of inter- and intraspecific genetic diversity, which is also important for determining species stability and for the conservation of distinct lineages. The genetically diverse but morphologically similar variants of cryptic species can be evaluated with COI barcodes, which helps in the potential discovery of new species or subspecies that might require conservation measures.

Until now, using DNA barcoding to document avian diversity has been limited primarily to North America, Korea, Argentina and Scandinavia [8, 9, 10, 11]. The main objective of this study is to utilize the DNA barcoding technique for the first time for birds in Turkey. This approach will help the characterization of the genetic diversity of bird species in this under-studied region and make it possible to compare this diversity to the COI barcodes of the same species living in different parts of the world. Combined with the available DNA barcodes from Scandinavia, Russia and Korea, comparisons that include Turkey will provide a better overview of the distribution of avian genetic diversity in the Palearctic. This study is also an important first step in preparing a database of genetic diversity for the species in the region, and will provide the baseline data for monitoring changes in species and genetic diversity over time. As the study location at Aras River wetlands is a crucial breeding, wintering and migration stopover site for at least 264 bird species, our findings are important because they can demonstrate variation in the genetic composition of widely-distributed bird species. The documentation of genetic diversity at this site is also significant for bird conservation because the government of Turkey is planning to build the Tuzluca dam on the Aras River that threatens to destroy this vital area where most of the bird species of Turkey have been recorded [12].

## Materials and Methods

**Fig 1:**
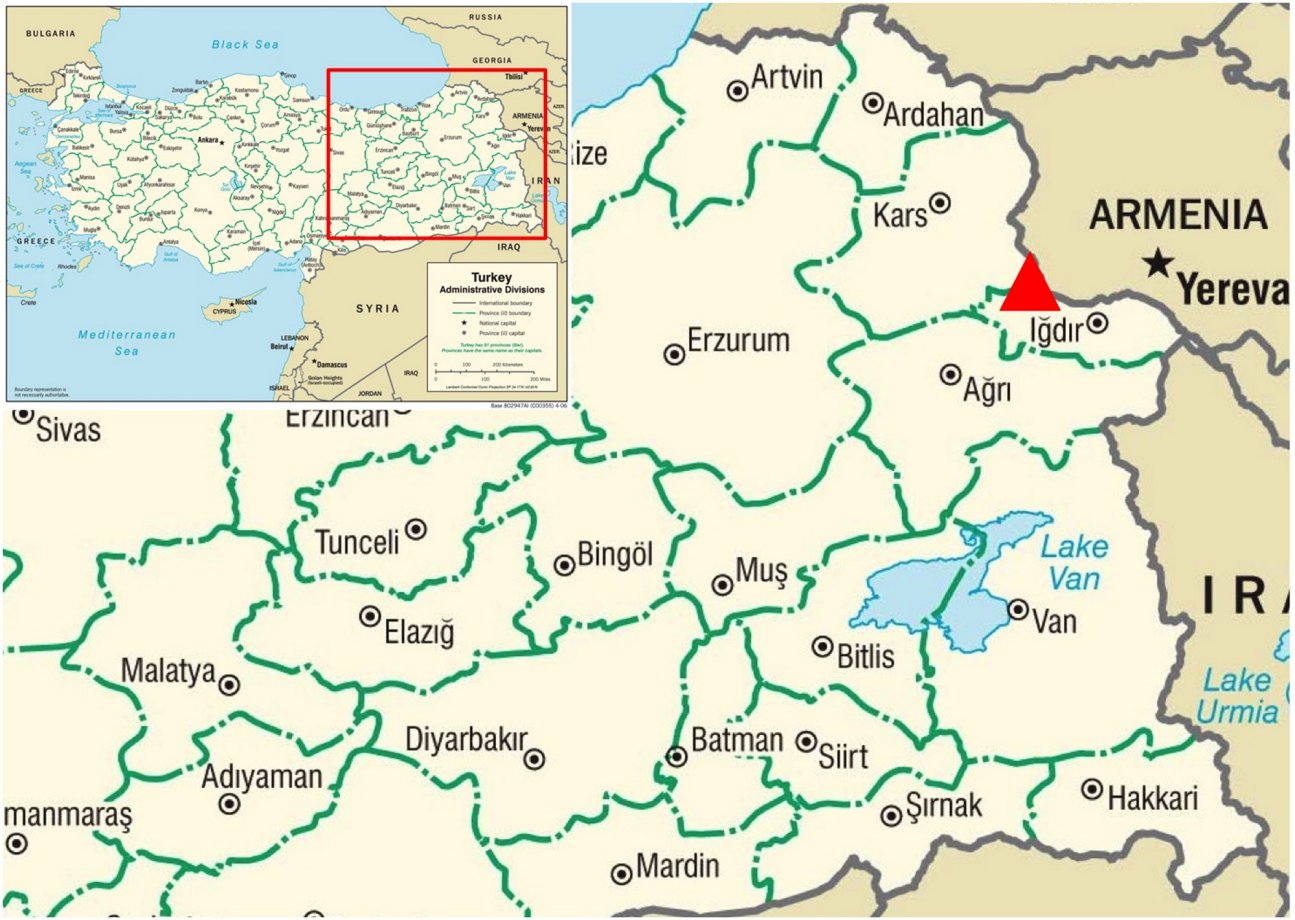
Map of the Aras River (Kars-Iğdır) biodiversity region and Aras Bird Research and Education Center bird banding (ringing) station.

The study was undertaken at the Aras River Bird Research and Education Center (Yukarı Çıyrıklı, Tuzluca, Iğdır; elevation: 960 m; 40°07'16''N 43°35'00'' E), the first bird banding station in eastern Turkey (Fig 1). At the foot of the 1500–2300 m high Kars plateau and surrounded by arid steppes of the Iğdır plains, the Aras River wetlands comprise a major migration stop-over, breeding, and wintering area for many bird species travelling between Africa, Asia, and Europe. These wetlands are at the confluence of Aras River and Iğdır Plains globally Important Bird Areas [2]. At the Aras River bird banding (ringing) station, over 75,000 individuals of 182 bird species have been banded and 264 species have been recorded since banding began in 2006, constituting 56% of the 473 bird species recorded from Turkey [13]. Birds were caught using 12 x 2.5 m Ecotone^™^ 1016 series mist nets designed for safe bird and bat sampling, with 16x16mm mesh made of D110 netting (DENIER 110/2) that capture birds without harming them. At the station, wetlands along the Aras River are sampled every year with mist nets, 40–60 of which are open every day between mid-March to early June and mid-August to early November. Nets are checked every half hour, birds are taken out, brought to the processing center, identified, banded, measured, and released after the collection of the blood sample. The bird community of Aras River wetlands is mostly typical of eastern Europe and the species examined for this study are common and well-known European species that were identified by licensed and experienced bird banders, using detailed field guides and identification books when necessary [14, 15, 16]. For this study, the collection of the blood samples was conducted in May 2009 and October 2009. Aras station has operated approximately 150 to 200 days every year since 2006. The birds sampled for this study are representative of the bird community at the site. All but two of the species analyzed in this study are captured every year and are among the most common 70% of the species captured. The species diversity and abundance during these seasons was comparable to, and did not differ significantly from, species diversities and abundances from other seasons (t-test, p = 0.062–0.83). Blood samples were collected following standard protocols. From the brachial vein of each bird, 20–50 μl blood was collected with a 30-gauge needle. Blood collected from each bird was less than 0.2% of its body weight, five times below the recommended maximum. No birds were injured or ill during the capture, handling and blood collection.

For extracting DNA from the blood samples, Roche High Pure PCR Template Preparation Kit (Germany) and Invitrogen Pure Ink Genomic DNA Extraction Kit (USA) were used, and the manufacturer protocols were applied. Cytochrome oxidase I gene (COI) was used as the molecular marker of choice, as it is accepted to be the unique DNA barcoding marker by the Consortium for the Barcode of Life (CBOL) and the All Birds Barcoding Initiative (ABBI) [17]. For PCR amplifications, the standard primers Bird F1 (TTCTCCAACCACAAAGACATT GGCAC), Bird R1 (ACGTGGGAGATAATTCCAAATCCTG), and Bird R2 (ACTACATGTGAGATGATTCCGAATCCAG) were used [11].

Protocols for COI amplification followed Hebert et al. [8]. For each PCR, 2 μl of DNA was added to a 48 μl reaction mixture. The mixture was composed of 4.5 μl of 10x high fidelity buffer (Fermentas), 4 μl of MgCl_2_ (25 mM), 1.5 μl of 10 mM deoxyribonucleotide triphosphate (dNTPs), 2.5 μl of each primer (20 μM), 0.3 μl of Taq DNA polymerase (5U/μl) and 32.7 μl H_2_O. Cycling parameters consisted of an initial denaturation step of 1 min at 94°C followed by 35 cycles of 1 min at 95°C, 1.5 min at 51°C and 1.5 min at 72°C, with a final extension step of 5 min at 72°C.

PCR products were cleaned up for further use in the sequencing reactions using Roche High Pure PCR Product Purification Kit (Roche, Mannheim). After the clean-ups, PCR products were sent to the Macrogen Inc. (South Korea) for commercial sequencing. The obtained base sequences were cleaned with the software Sequencher v. 4.1 (Gene Codes Corp.), and Clustal X [18] was used for the sequence alignments. All obtained sequences were submitted to the Consortium for the Barcode of Life database (BOLD) (process ids ARIGB001-16—ARIGB00170-16) and GenBank (accession numbers KX283100-KX283167).

A neighbor-joining tree for each species was prepared with the samples sequenced in this study, combined with the sequences obtained from BOLD for each species, using Kimura 2-parameter distances in the software MEGA 4.0 [19]. TCS 1.21 [20] was used to prepare haplotype networks. Intraspecific and interspecific distances were calculated with MEGA 4.0, again, using Kimura 2-parameter distances.

## Results

**Fig 2:**
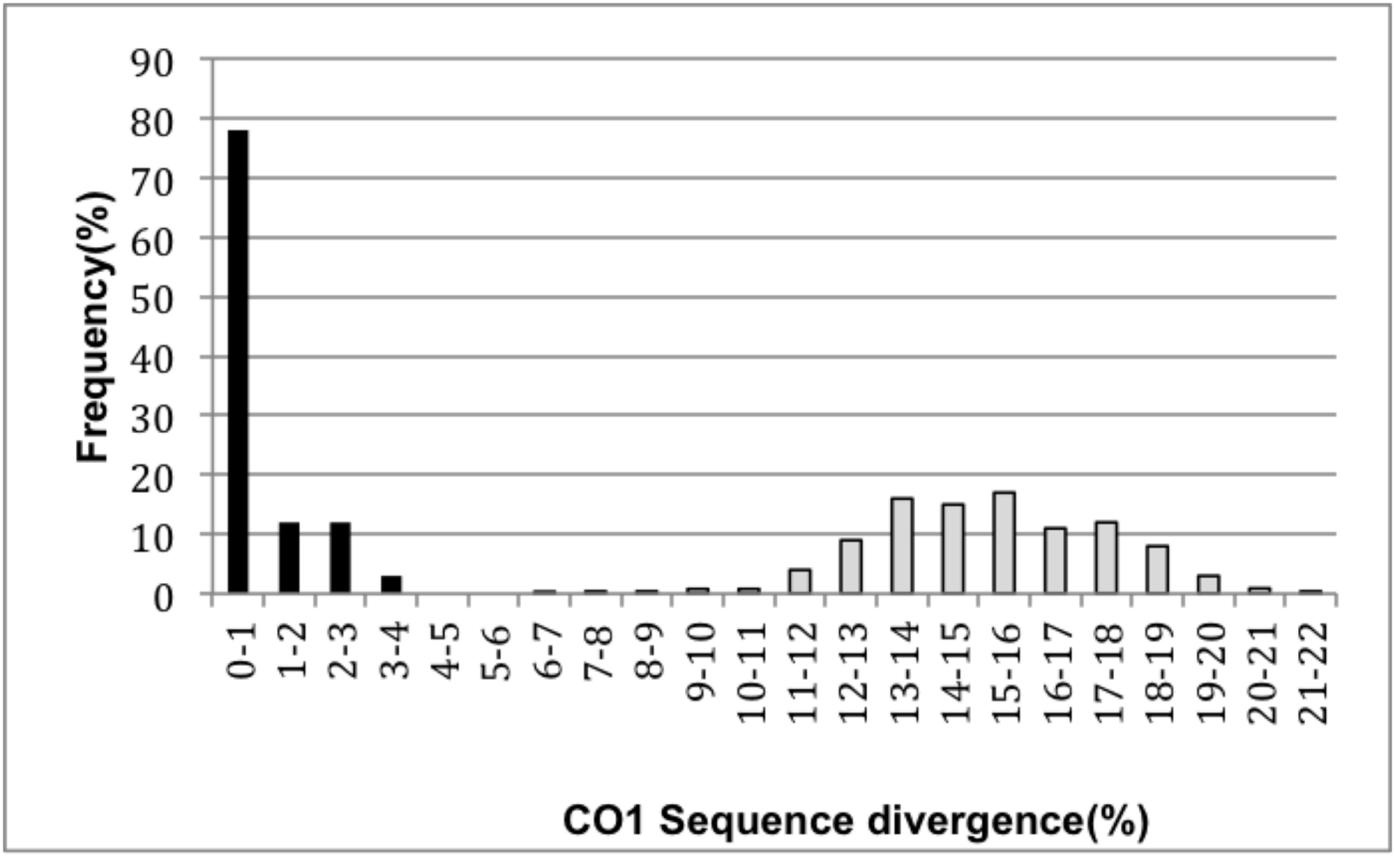
Frequency distribution of the mean divergences for COI sequences (Kimura 2- parameter model) of the 73 samples. Two taxonomic levels are represented: species (dark bars) and genus (gray bars).

As a result of our analyses, we generated 73 COI sequences from 33 different species (Table 1, S1 Table). These 33 species belonged to 26 different genera. The mean intraspecific divergence was 0.62%. However, we observed relatively high intraspecific divergence values in five cases: *Sylvia curruca* 3.2% (based on 12 sequences), *Saxicola maurus* 2.8% (based on eight sequences), *Phoenicurus phoenicurus* 2.6% (based on 13 sequences), *Parus major* 1.7% (based on 21 sequences), and *Caprimulgus europaeus* 1.7% (based on six sequences). When these clades were removed, the mean intraspecific divergence dropped to 0.3%. The minimum interspecific distance was 6.8% (Fig 2).

**Table 1:** List of similarity percentages of group I species.

	Closest Match	Second Closest Match	Third Closest Match
*Coturnix coturnix*	100% with *Coturnix coturnix*	99.59% with *Coturnix coturnix*	97.94% with *Coturnix japonica*
*Cuculus canorus*	100% with *Cuculus canorus*	99.53% with *Cuculus canorus*	99.53% with *Cuculus optatus*
*Ficedula parva*	100% with *Ficedula parva*	94.54% with *Ficedula albicilla*	94.34% with *Ficedula parva*
*Oenanthe hispanica*	100% with *Oenanthe hispanica*	99.82% with *Oenanthe pleschanka*	99.64% with *Oenanthe pleschanka*
*Emberiza citrinella*	100% with *Emberiza citrinella*	99.77% with *Emberiza citrinella*	99.77% with *Emberiza leucocephalos*
*Emberiza hortulana*	99.4% with *Emberiza hortulana*	99.2% with *Emberiza caesia*	95.21% with *Emberiza buchanani*

For 12 species, 18 new haplotypes were recorded for the first time. Three new haplotypes were observed in *Acrocephalus palustris*, two new haplotypes each were observed in *Alcedo atthis*, *Emberiza hortulana* and *Phoenicurus phoenicurus*, and one new haplotype each was observed in *Emberiza schoeniclus*, *Ficedula parva*, *Locustella luscinioides*, *Oriolus oriolus*, *Passer domesticus*, *Phylloscopus trochilus*, *Saxicola maurus*, *Saxicola rubetra* and *Sylvia curruca*.

**Fig 3:**
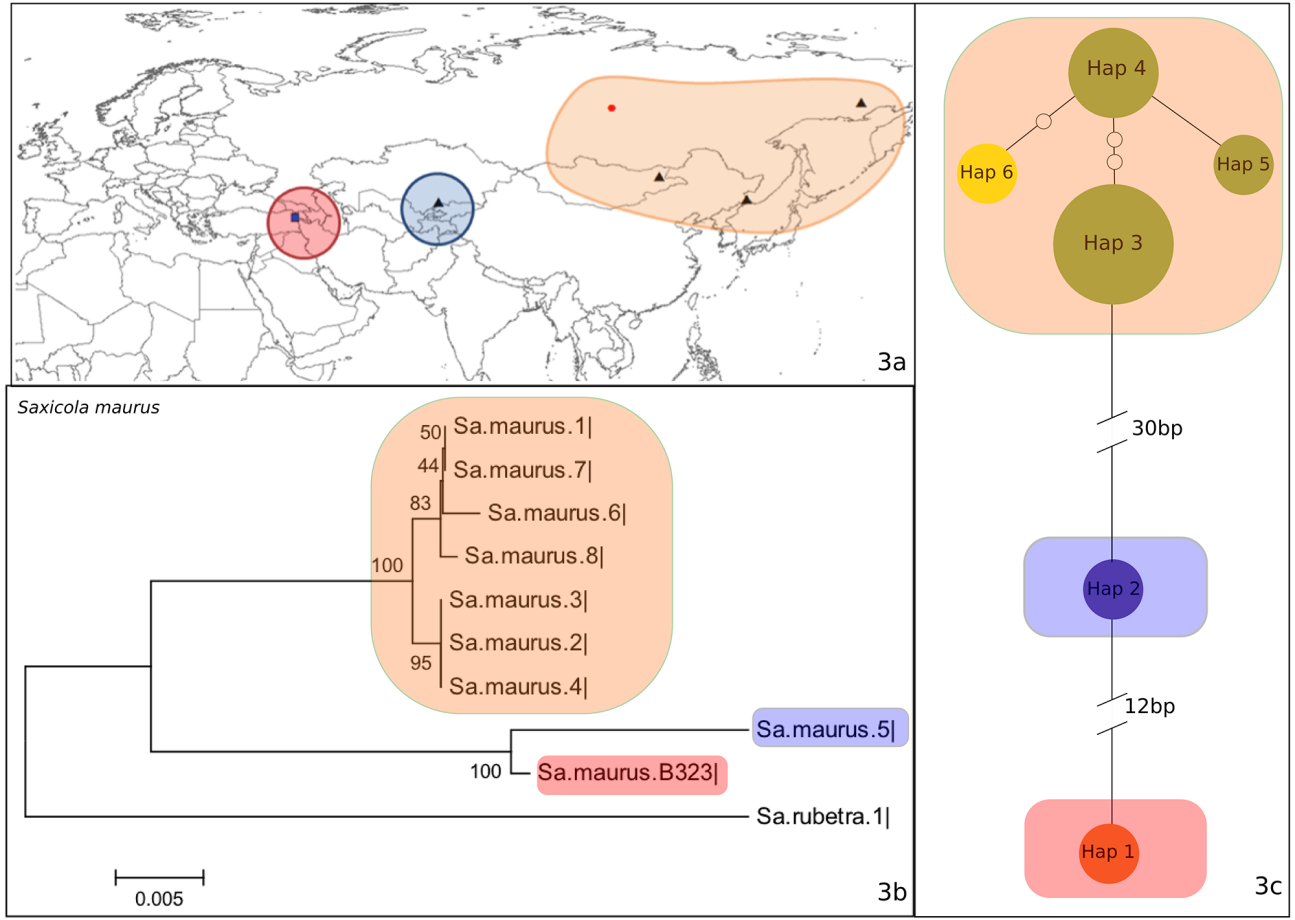
a) The locations for which COI Barcode Data were available from BOLD for *Saxicola maurus* (Group I). The black triangles indicate localities with GPS coordinates, the red circles indicate countries for which GPS data were not available, and the blue squares indicate the study site (Aras River Research Station). Red area corresponds to the distribution area of Hap 2, blue area to Hap 1, and orange area to Hap 3, 4, 5, and 6. b) Neighbor joining tree. c) Haplotype network, where colors indicate the origin of the haplotypes (Green: Russia, Purple: Turkey, Yellow: Mongolia, Orange: Kazakhstan).

26 of the 33 bird species analyzed in this study had unique barcode sequences that were distinct from those found in any other species in the BOLD. Seven species had shared or overlapping barcode sequences with other congeneric species. These species, categorized as group I, are *Coturnix coturnix* (S1a Fig), *Cuculus canorus* (S1b Fig), *Ficedula parva* (S1c Fig), *Oenanthe hispanica* (S1d Fig), *Emberiza citrinella* (S1e Fig), *Emberiza hortulana* (S1f Fig), and *Saxicola maurus* (Fig 3).

In order to illustrate the basic pattern in group I, we present the results from *Saxicola maurus* in greater detail (Fig 3). In this species, nine different samples from Russia, Mongolia, Kazakhstan and Turkey were compared with the one sample collected from the Aras River Research Station (Fig 3a). A comparison with the BOLD showed that the two closest matches of the Aras sample were to *Saxicola maurus* and *Saxicola torquatus* with 99.35% similarity, and the third to *Saxicola maurus* with 99.19% similarity. *Saxicola rubetra* was used as an outgroup in the neighbor joining tree (Fig 3b). In this tree, the Aras sample (B323) and Kazakhstan sample (*S*. *maurus* #5) formed a separate clade, apart from the rest of the samples. Six different haplotypes were observed for this species, which clustered into three main groups in the haplotype network (Fig 3c). These groups are in Aras, central Asia and eastern Asia (Fig 3a). Comparisons of the other six species in group I also show high levels of matches to other species in the BOLD (Table 1).

**Fig 4:**
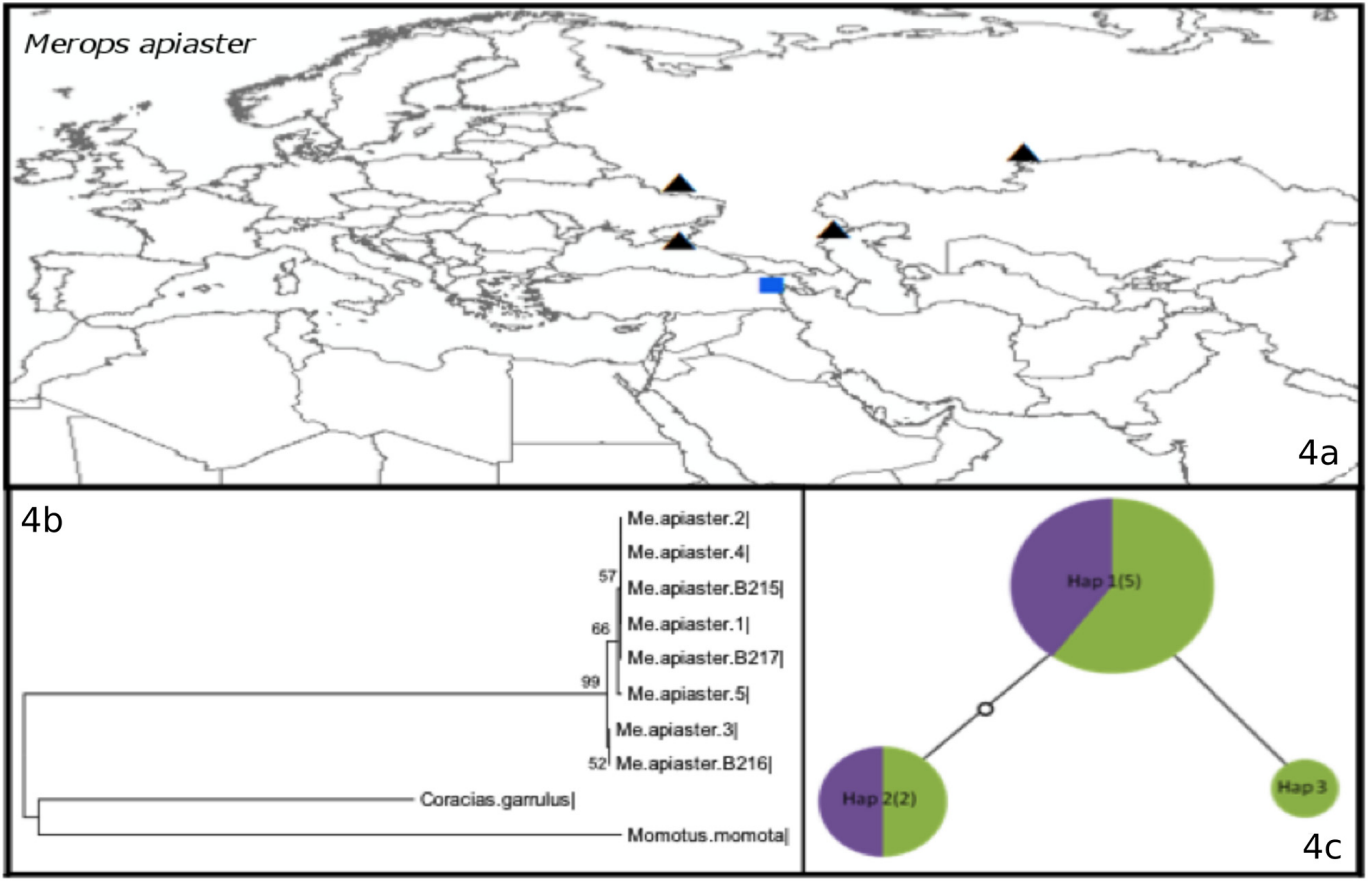
a) The locations for which COI Barcode Data were available from BOLD for *Merops apiaster* (Group II). The black triangles indicate localities with GPS coordinates, the red circles indicate countries for which GPS data were not available, and the blue squares indicate the study site (Aras River Research Station). b) Neighbor-joining tree. c) Haplotype network, where colors indicate the origin of the haplotypes (Green: Russia, Purple: Turkey).

Our analyses revealed three additional groups based on intraspecific divergence, taxonomy, and barcoding suitability (*i*.*e*. whether the species had species specific DNA barcodes in BOLD or not). Group II is composed of five species (*Lanius minor* (S2a Fig), *Acrocephalus palustris* (S2b Fig), *Saxicola rubetra* (S2c Fig), *Emberiza calandra* (S2d Fig), and *Merops apiaster* (Fig 4), which had no recognized subspecies, showed no intraspecific divergence and had unique DNA barcodes. For these species, all Aras samples clustered closely with the rest of the barcodes from the BOLD. A good example of the pattern in group II is *Merops apiaster*, for which eight samples from Russia and Turkey were analyzed (Fig 4a). The Aras samples (B215, B216, and B217) clustered closely with the rest of the barcodes from the BOLD (Fig 4b). Three different haplotypes were observed for *Merops apiaster* (Fig 4c), differentiated from each other by a maximum of two bp.

**Fig 5:**
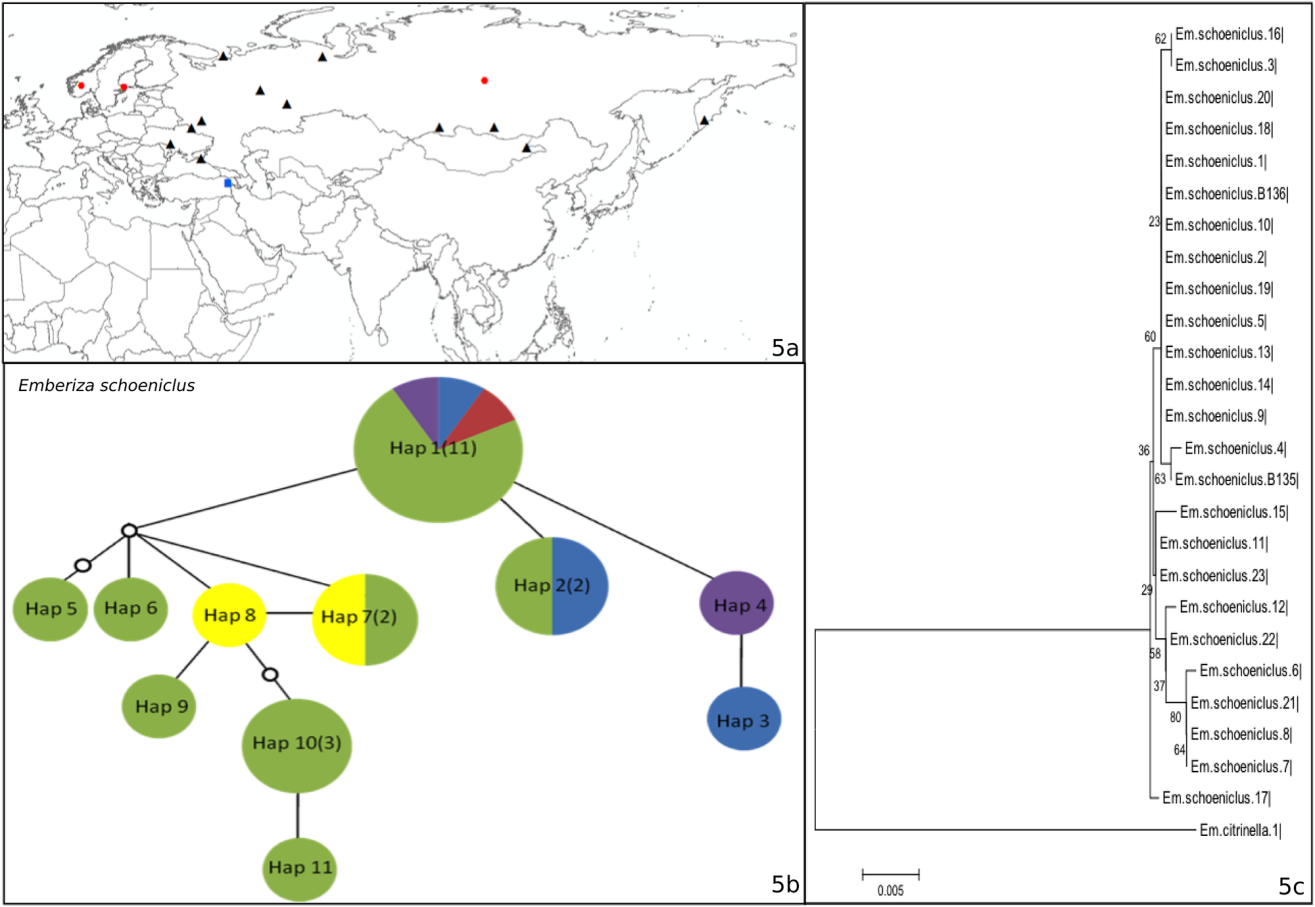
a) The locations for which COI Barcode Data were available from BOLD for *Emberiza schoeniclus* (Group III). The black triangles indicate localities with GPS coordinates, the red circles indicate countries for which GPS data were not available, and the blue squares indicate the study site (Aras River Research Station). b) Haplotype network, where colors indicate the origin of the haplotypes (Green: Russia, Purple: Turkey, Yellow: Mongolia, Blue: Sweden, Red: Norway). c) Neighbor-joining tree.

Group III contained 16 species, *Alcedo atthis*, *Coracias garrulus*, *Oriolus oriolus*, *Galerida cristata*, *Remiz pendulinus*, *Cettia cetti*, *Phylloscopus trochilus*, *Locustella luscinioides*, *Sylvia nisoria*, *Muscicapa striata*, *Luscinia svecica*, *Turdus merula*, *Sylvia atricapilla*, *Passer domesticus*, *Passer montanus* (S3a–S3o Fig), and *Emberiza schoeniclus* (Fig 5). These species, similar to those in Group II, showed no intraspecific divergence and had unique DNA barcodes, but had taxonomically defined subspecies within their ranges.

*Emberiza schoeniclus* (Fig 5) demonstrates the pattern in group III. Eleven different haplotypes were observed in the network for this species, all with very little differentiation (Fig 5b). Twenty five different samples from five different countries (Russia, Turkey, Mongolia, Sweden, and Norway) were analyzed for this species, along with two samples collected from Aras (B135, B136; Fig 5a). *Emberiza schoeniclus* formed a monophyletic group, including the samples from Aras (Fig 5c). Hap 1 was the most common haplotype, observed in eleven different samples from Russia, Sweden, Turkey and Norway. Hap 4 was a new haplotype that was recorded only in Turkey.

**Fig 6:**
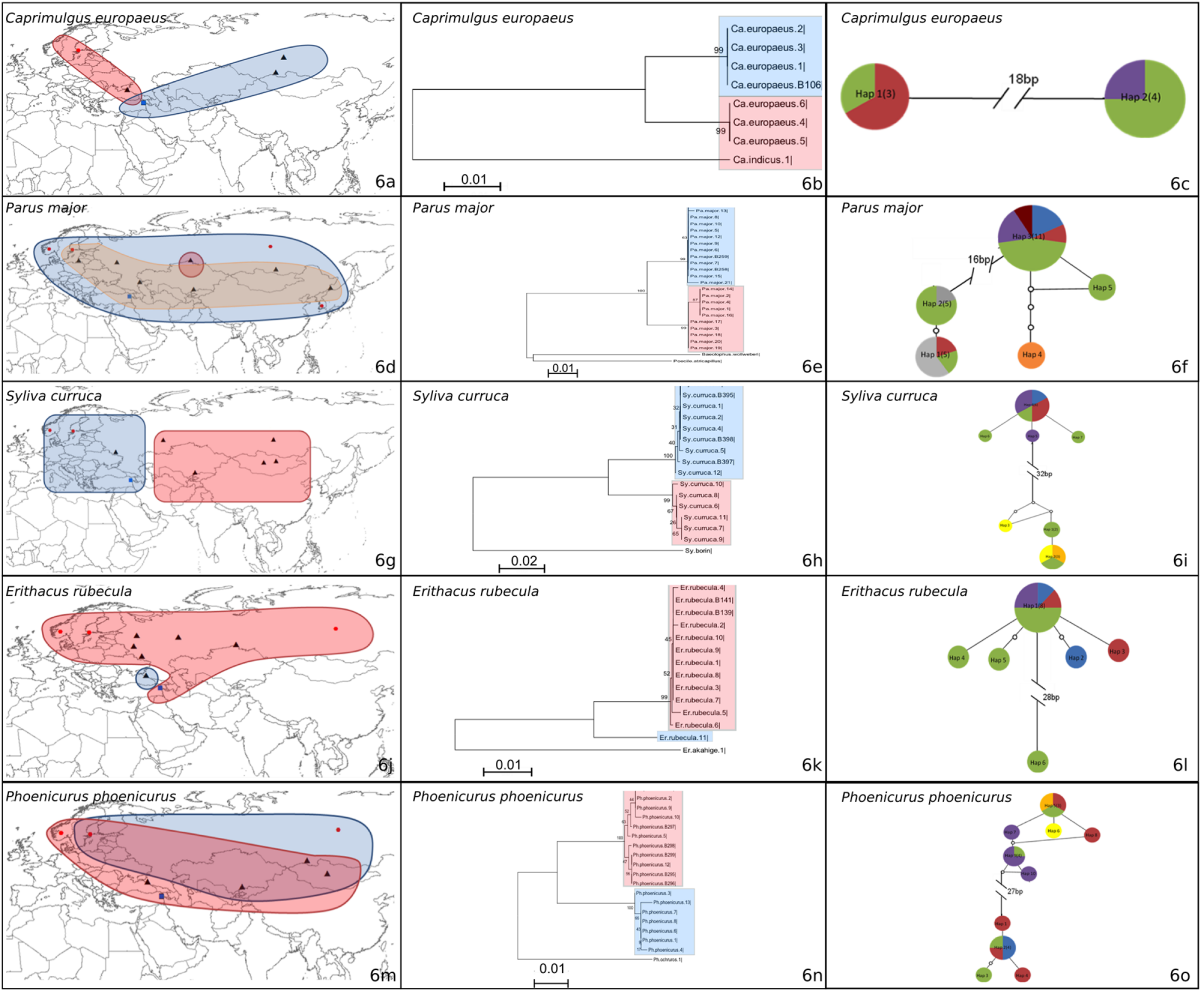
a) The locations for which COI Barcode Data were available from BOLD for Group IV. The black triangles indicate localities with GPS coordinates, the red circles indicate countries for which GPS data were not available, and the blue squares indicate the study site (Aras River Research Station). For the haplotype networks, colors indicate the origin of the haplotypes (Green: Russia, Purple: Turkey, Yellow: Mongolia, Blue: Sweden, Red: Norway, Grey: South Korea, Dark Red: Lithuania). b) Neighbor-joining trees. c) Haplotype networks. For *Caprimulgus europaeus*, red and blue areas indicate Hap 1 and Hap 2 respectively. For *Parus major* red, orange and blue areas indicate Hap 3, 4, 5, and Hap 1, 2, respectively. For *Sylvia curruca*, Hap 1, 2, 3 are indicated by red and Hap 4, 5, 6, 7 are indicated with blue areas. For *Erithacus rubecula*, blue area indicates Hap 6 and other haplotypes’ geographical distributions are indicated with red. For *Phoenicurus phoenicurus*, Hap 5, 6, 7, 8, 9, 10 are indicated with red and Hap 1, 2, 3, 4 are indicated with blue.

Group IV consists of five species (*Caprimulgus europaeus*, *Parus major*, *Sylvia curruca*, *Erithacus rubecula*, and *Phoenicurus phoenicurus*) which have designated subspecies in their ranges, show high intraspecific divergence and DNA barcodes that were different from those found in other species (Fig 6). For each species, we provide a tree and a haplotype network showing the differentiation of the clades and a map showing the geographic distribution of these clades. Seven different samples from three different countries (Russia, Turkey, and Norway) were analyzed for *Caprimulgus europaeus*, along with one Aras sample (Fig 6a). For this species, two different haplotypes were observed, also confirmed by the existence of two clades in the neighbor-joining tree (Fig 6b and 6c). These two haplotypes were separated by 18 base pairs. Hap 1 (red) was observed in Norway and Russia, whereas Hap 2 (blue) was observed in Turkey and Russia (Fig 6a). The distribution map shows that Hap 1 is common in the western parts of the species’ range whereas Hap 2 is more common in the eastern parts.

For *Parus major*, 23 different samples from seven different countries (Russia, Turkey, Lithuania, Norway, Kazakhstan, South Korea, and Sweden) were analyzed, along with two samples collected from Aras (Fig 6d). *Parus major* samples formed three main clades, with the samples from Turkey (B258 and B259) in one of these groups (Fig 6e). Five different haplotypes were observed in this species (Fig 6f). The most common haplotype, Hap 3, was found in 11 different samples from five countries (Aras, Russia, Sweden, Norway and Lithuania). This was different from Hap 1 and Hap 2 by 16 bp. Hap 4 (red) was found in a single location in Russia, whereas Hap 3 and Hap 5 (orange), and Hap 1 and Hap 2 (blue) were found almost sympatrically in the entire distribution of the barcoded individuals for this species (Fig 6f).

Fifteen different samples from Russia, Turkey, Mongolia, Kazakhstan, Sweden, and Norway, including three from Aras, were analyzed for *Sylvia curruca* (Fig 6g), also in group IV. *Sylvia curruca* formed two clades in the neighbor-joining tree, and the Aras samples (B335, B337 and B338) clustered in the same clade (Fig 6h). Different clades indicated the eastern and western parts of this species’ range. This differentiation of the haplotypes into two clades (a 32 bp difference) in the neighbor-joining tree was also reflected in the presence of two haplotype groups in the *Sylvia curruca* network (Fig 6i). Specifically, the first group contained nine samples from Russia, Turkey, Norway, and Sweden, comprising the ‘western clade’. The ‘eastern’ group was composed of six samples from Russia, Kazakhstan, and Mongolia.

Fifteen samples from four different countries (Russia, Turkey, Sweden and Norway) were analyzed for the fourth species in group IV, *Erithacus rubecula*, including two samples from Aras (Fig 6j). Again, two main groups were observed in the haplotype network. All Aras samples (B139, B141) clustered in the first group (red), whereas the second group contained only one sample from Russia (blue; Fig 6j). Hap 6, found in Russia, was highly divergent from the other samples by 28 bp (Fig 6k and 6l).

Eighteen different samples from Russia, Turkey, Kazakhstan, Mongolia, Norway and Sweden were analyzed for *Phoenicurus phoenicurus* (Fig 6m), including five samples from Aras. The 10 different haplotypes observed clustered into two groups, with a 27 bp difference in the haplotype network (Fig 6n and 6o). The first group (blue; Fig 6m) was composed of 11 samples from Russia, Turkey, Norway, Mongolia and Kazakhstan, and the second group (red) contained seven samples from Russia, Norway, and Sweden.

## Discussion

The main objective of DNA barcoding is to help with the identification of unknown specimens and facilitate the discovery of new taxa. To be able to undertake these objectives using the DNA barcoding approach, a threshold is needed. This threshold should be high enough to separate specimens that belong to different species and low enough to recognize recently diverged species. Hebert et al. [8] proposed a threshold to define new species, which he called the "barcoding gap", defined as ten times the mean intraspecific variation for the studied group. In this study, the mean intraspecific divergence was 0.62% for the samples analyzed, and the resulting barcoding gap of 6.2% is indeed less than the lowest observed interspecific distance of 6.8%. Hence, for the species in our study, the barcoding gap worked effectively. A distribution of intra- and interspecific divergence frequencies is given in Fig 2. A study on avian diversification showed that the average intraspecific distance was 0.32% for the Palearctic dataset [21], dropping to 0.19% if highly variable species are excluded. For the Aras samples, the exclusion of highly variable species dropped the average intraspecific distance to 0.30%. A study on North American birds [8] indicated that some species have deep intraspecific divergences that are 9- to 17-fold higher than the average distances, whereas the most divergent Aras samples had intraspecific divergence values 8-fold higher.

The patterns observed in the 33 species define four main groups. The first group consists of the seven species for which DNA barcodes did not uniquely define species. These likely represent cases of mitochondrial introgression between species, which has been previously documented in birds [22]. The DNA barcodes of *Coturnix coturnix* and *Ficedula parva* had high overlap with species that were previously recognized as their subspecies [23]. The DNA barcode of *Coturnix coturnix* had 97.94% similarity with that of *Coturnix japonica* and the DNA barcode of *Ficedula parva* had 94.52% similarity with that of *Ficedula albicilla*. Although we accepted *Coturnix japonica* as a distinct species in this study, there is disagreement about recognizing it as a distinct species [23]. Accepting *Coturnix japonica* as a distinct species meant that no intraspecific divergence was observed in *Coturnix coturnix* samples from Russia, Sweden, and Turkey. On the other hand, *Ficedula parva* samples had a mean intraspecific divergence of 1.6% as a result of the divergent South Korean samples. The *Ficedula parva* sample from South Korea clustered into the *Ficedula albicilla* clade. Since *Ficedula albicilla* is an Asian taxon and is still recognized as *Ficedula parva albicilla* by some authorities [24], it is possible that the South Korean sample retrieved from the BOLD may actually be *Ficedula albicilla*, instead of *Ficedula parva*. Regardless, the non-overlapping distribution [25] and large divergence of the eastern and western haplotypes suggest long-term isolation between these taxa.

Another member of the first group that was not suitable for barcoding is *Cuculus canorus*, since its DNA barcode had 99.53% similarity with that of *Cuculus optatus*. This species also had large intraspecific divergence, with a mean of 1.4%. *Cuculus canorus* is a brood parasite so lays its eggs in the nests of other bird species. Since cuckoos use different species as hosts, they evolve different gentes to mimic eggs of hosts. As a result, the risk of the eggs being rejected by the hosts is reduced. Gentes are restricted to female lineages; since males are genetically identical, the common cuckoo remains genetically as one species. Studies [26] show that there is differentiation between gentes in maternally inherited mitochondrial DNA, but not in microsatellite loci of nuclear DNA. The causes of the shared mitochondrial haplotypes between these species are not yet determined and hybrids have never been documented [26].

A different pattern in the first group was observed in the Emberizidae family and in *Oenanthe hispanica*. Sister species from Emberizidae family were phenotypically distinct but their mitochondrial DNA is similar [10]. The DNA barcodes of *Emberiza citrinella* and *Emberiza leucocephalos* showed 99.77% similarity with each other. These species breed across western and central Siberia, with *Emberiza citrinella* extending to Western Europe and *Emberiza leucocephalos* extending to the Far East. Although two species differ phenotypically, they become more similar across their sympatric ranges, which suggests that they could be hybridizing [27]. *Emberiza hortulana* and *Emberiza caesia* are also phenotypically distinct sister species with 99.2% similarity in the COI regions. There is no evidence for the hybridization of *Emberiza hortulana* and *Emberiza caesia* and their ranges hardly overlap [28]. Studies indicate that the cytochrome-b sequences are more similar in the two sister pairs *Emberiza leucocephalos*–*Emberiza citrinella* and *Emberiza hortulana*–*Emberiza caesia* than in other sister species. Similarly, *Oenanthe hispanica* and its sister species *Oenanthe pleschanka* had DNA barcodes that were 99.82% similar. The main reason for this similarity could again be introgression between the two species [22].

A different example of the pattern observed in the first group is seen in *Saxicola maurus*, with its 99.35% DNA barcode similarity to *Saxicola torquatus*. This species had three different haplotypes, which are from three distinct geographical areas: Turkey, Central Asia and Eastern Asia. Although Turkey is not geographically in between Eastern and Central Asia, the Turkish haplotype was between the Asian haplotypes in the haplotype network. These three haplotypes may belong to three different subspecies since the ranges of subspecies were consistent with the ranges of haplotypes. In this case, the Turkish haplotype could represent *Saxicola maurus armenicus* since this subspecies is found in the mountains of eastern Turkey to Transcaucasia and Iran. Haplotypes from Central Asia possibly belong to *Saxicola maurus maurus* since this subspecies is found in East Russia to central Asia, and the third haplotype might be *Saxicola maurus stejnegeri*, with a range covering East Siberia to Japan and Korea. This pattern is parallel to that reported by Illera et al. [29] and Zink et al. [30] in *Saxicola*, and corroborates their findings.

In group II, *Acrocephalus palustris*, *Emberiza calandra* and *Saxicola rubetra* had six, three and five haplotypes, respectively. Samples were from Turkey, Russia, Norway and Sweden. Although three new haplotypes were recorded for *Acrocephalus palustris*, all haplotypes were nearly identical. For *Lanius minor* and *Merops apiaster*, the different haplotypes had only two or three bp differences. This result confirms the absence of subspecies in these species, since there was no genetic divergence between the samples collected from different locations.

All members of group III have several subspecies, and these subspecies are generally defined according to the differences in morphology like size, bill size or plumage color. For instance, there are seven subspecies recognized for *Alcedo atthis*, and samples from Russia, Turkey, Sweden, Norway, Kazakhstan, Mongolia, and South Korea were analyzed in this study. Although European and Korean samples belong to different subspecies, no significant intraspecific genetic variance was observed in the DNA barcodes. Other studies also confirm the absence of genetic variability between *Alcedo atthis* subspecies [31]. Similarly, there are five recognized subspecies for *Sylvia atricapilla*, seven for *Muscicapa striata*, nine for *Passer montanus*, 11 for *Luscinia svecica*, 15 for *Turdus merula*, 16 for *Emberiza schoeniclus*, and 37 for *Galerida cristata* (three of these subspecies, *Galerida cristata caucasica*, *Galerida cristata subtaurica*, and *Galerida cristata zion* are observed in Turkey [32]. Samples from an extensive area, including Russia, Turkey, Mongolia, Kazakhstan, South Korea, Sweden and Norway, were analyzed for these species. Although these countries cover the ranges of many subspecies, all COI sequences were nearly identical within each species. Haplotypes for each species differed by only one to eight bp. Since the subspecies of these species are mainly defined by color or size differentiation, the genetic similarity and the lack of genetic differentiation among these different subspecies is interesting, suggesting that phenotypic differences are not always reflected in differences in mtDNA. It should be noted that mtDNA goes through selective sweeps and bottlenecks frequently [33], and the lack of differentiation based on mtDNA alone does not necessarily mean a lack of evolutionary divergence.

A subset of the third group consists of *Locustella luscinioides* and *Phylloscopus trochilus*. Both of these species have three recognized subspecies. Four *Locustella luscinioides* samples from Turkey and Sweden were analyzed. Samples from Turkey (subspecies *L*. *l*. *fusca*), were differentiated from the Swedish samples (*L*. *l*. *luscinioides*). Similarly, the DNA barcode differentiation of *Phylloscopus trochilus* samples we analyzed was in concordance with the accepted subspecies designations. According to our study, samples from both countries had similar DNA barcodes.

In group III, the COI sequences for *Passer domesticus* samples from Russia, Turkey, Argentina, Norway, Kazakhstan, Canada, and Sweden analyzed in this study, a maximum of six base pair differences were observed, although 12 subspecies are recognized for this species. Being an introduced species might be the main reason behind the similarity in the DNA barcodes for the North American samples. Although the native range of the house sparrows contains most of Europe and Asia, this species is now found on every continent except Antarctica. Even though house sparrows exhibit broad phenotypic divergence in body mass, sexual dimorphism and metabolic rate in both their native and introduced ranges, DNA barcodes analyzed in this study and others are highly similar [34]. DNA barcode for species such as *Locustella luscinioides* or *Passer domesticus* can distinguish them from other species, but are not useful for genetically diagnosing subspecies.

For another species in the third group, *Cettia cetti*, although three recognized subspecies exist, the analyzed samples from Russia and Turkey had identical DNA barcodes. These samples may belong to *Cettia cetti orientalis* or *Cettia cetti cetti*, since these subspecies are observed both in Turkey and Russia. Another species in group III, *Coracias garrulus* has two subspecies in its range. *Coracias garrulous garrulus* was found in North Africa, Europe to Iran and southwest Siberia and *Coracias garrulus semenowi* was found in Iraq to west Xinjiang and south Kazakhstan. Both subspecies winter in South Africa but in distinct locations [35]. For this species, samples from Russia, Turkey, Sweden, and Kazakhstan were analyzed. Again all samples had nearly identical COI sequences. In these cases, it was harder to state the absence of genetic divergence between subspecies, since all analyzed samples may belong to the same subspecies, and there was no previously available genetic data for different subspecies to use as a basis for diagnosis.

Also in the third group were species for which all the barcodes in the BOLD most likely came from a single subspecies. Both *Oriolus oriolus* and *Sylvia nisoria* have two subspecies in their ranges. For *Oriolus oriolous*, one of the subspecies is observed in the eastern part of its range from west Siberia to the Indian subcontinent, whereas the other occurs in the western portion, from Europe to the Ural Mountains. All samples that were analyzed in this study were from the western subspecies, *O*. *o*. *oriolus*. Similarly, for *Remiz pendulinus*, all samples analyzed in this study belonged to *R*. *p*. *pendulinus*. As expected, all analyzed DNA barcodes were very similar to each other for these species, as they represent samples from only one subspecies for each species examined.

All members of group IV had several genetically divergent subspecies with unique DNA barcodes. For both *Caprimulgus europaeus* (six subspecies) and *Sylvia curruca* (two subspecies), two main divergent and geographically isolated haplotype groups were observed. First haplotype group was common in the western parts of the species’ range, whereas the second group was common in the east. Therefore, these species exhibit an allopatric population system. Mean intraspecific divergences were 1.7% and 3.2% for *Caprimulgus europaeus* and *Sylvia curruca*, respectively.

In *Erithacus rubecula*, despite covering the ranges of different subspecies, there were only two haplotypes. The samples from Russia, Sweden, Turkey, and Norway were genetically different from the sample from Russia. The mean intraspecific divergence was 0.9%.

A different pattern in group IV was observed for *Parus major* and *Phoenicurus phoenicurus*. Three and two main different haplotype groups were observed for *Parus major* and *Phoenicurus phoenicurus*, respectively. These different haplotypes had overlapping ranges. Mean intraspecific divergence was 1.7% for *Parus major* and 2.6% for *Phoenicurus phoenicurus*. The most common haplotype of *Parus major* was widely distributed, and observed in Sweden, Norway, Russia, Lithuania, and the study site (Aras). According to Kvist et al. [36], this observation supports a scenario of a recent range expansion, which can be explained by the confinement of this species to a single refugium during the Pleistocene. After the end of this period, the species spread to new habitats. For the case of *Phoenicurus phoenicurus*, genetic lineages appear to be largely sympatric [10], since there is a 26 base pair difference even among the Norwegian samples. Such a range overlap of two divergent clades might suggest the presence of two species, rather than subspecies, and detailed taxonomic evaluation of individuals from each clade might help elucidate the issue.

The first barcoding study of birds in Turkey showed that the bird community of Aras River wetlands, 1650 kilometers east of Bulgaria and 5 km west of Armenia, has mostly an eastern European-western Russian genetic signature, a result of its important location at the intersection of three major migratory flyways from western Palearctic to Middle East, India and Africa [37]. Even though our barcoding results mostly agreed with morphological/phenotypic species identifications, our findings from seven species in group I show that even for some “well-known” and morphologically distinct European bird species, molecular barcoding results may not agree with phenotypic species identifications based on morphology. The fact that molecular barcoding results in 21% (seven out of 33) well-known European bird species did not distinguish them sufficiently from similar species suggests that: 1-the genetic sequence used for molecular barcoding may not be diverse enough to accurately identify every species in a community; 2- hybridization, introgression, and shared mitochondrial haplotypes in some species limit the effectiveness of barcoding, and 3-barcoding data should not be relied upon exclusively for identifying bird species and need be interpreted in light of other genetic, morphological and vocal analyses, especially given that molecular barcoding is still evolving and many bird species have not been studied with this method. Especially in bird banding studies and other research that involves the capturing of birds, we recommend that tissue samples (ideally blood, if not at least feathers) are taken from every individual to be barcoded and the birds are morphologically identified to subspecies if possible. If this were done for every bird that is banded, every year we would be able to barcode millions of individuals of thousands of bird species. The resulting increase in our knowledge would tremendously improve our understanding of avian phylogenies, would reveal new cryptic bird species, and would help resolve much of the discrepancies currently observed between species identifications based on morphology and phenotypes versus molecular barcoding and genotypes.

DNA barcoding can also help to identify patterns of migratory connectivity for populations breeding in unstudied areas. Many of the species that pass through the Aras banding station are on their way to remote breeding grounds in Russia and Siberia. Because breeding ecology information from this area is so limited, DNA samples from en route migrants can be used to establish patterns of genetic similarity. The degree of genetic similarity can then be used to infer distinct breeding populations and migratory connectivity.

The gathering of the DNA barcoding data also made it possible to have a snapshot of the genetic diversity in the region before the possible building of the intended Tuzluca dam on the Aras River [12] that would destroy this critical migratory stopover, breeding and wintering site if the efforts to stop the dam fail [13]. Turkey has already lost 1.3 million hectares of its wetlands since the 1950s and the recent dismantling of Turkey’s environmental laws have [38] enabled the destruction of many remaining wetlands in the past decade. Over 75,000 birds from dozens of countries on three continents have been banded at Aras River wetlands and millions of birds likely use this riparian oasis. If constructed, the Tuzluca dam will wipe out vital habitat for this international bird community. The data collected in this study will also make it possible to document the impact of the Tuzluca dam on the genetic composition of the new bird community if Government Hydraulic Works (DSI) of Turkey ends up destroying the globally important Aras River Bird Paradise, home to at least 264 bird species at the confluence of three global migratory flyways [37].

## Conclusion

For the first barcoding study on Turkey’s birds, we applied genetic barcoding to birds captured in the Aras River wetlands on the Kars-Iğdır border, and compared our findings to the previous barcoding studies at continental scales. Our results generally agreed with the DNA barcoding literature both in terms of the effectiveness of COI barcodes as an identification tool and the existence of a barcoding gap [8]. Samples from five different orders, Passeriformes, Cuculiformes, Galliformes, Coraciiformes, and Caprimulgiformes were studied. From 12 species, 18 new haplotypes were recorded from the Aras samples. Unique barcodes/haplotypes were found in 26 of the 33 studied species. Four main groups were identified based on barcoding suitability, current subspecific classification and levels of intraspecific divergence at continental scales. Group I was composed of five species, which have no defined subspecies and no intraspecific divergence, and Group III consisted of five species which had designated subspecies with high intraspecific divergence. Hence these 10 species in groups I and III show concordance between subspecific taxonomic classifications and genetic differentiation. On the other hand, Group II had the greatest number of species in it, 16, which had taxonomically defined subspecies, but no corresponding intraspecific divergence. This suggests that in these species, at least some subspecific classifications are not supported by genetics and there might be over-splitting in their taxonomy. Finally in Group IV, we observed five species that had several genetically divergent subspecies with unique DNA barcodes. Our study shows how data from local scales and seemingly independent DNA barcoding studies can be used together to make phylogeographical comparisons at continental scales and taxonomical inferences. We recommend that this approach also be used with other groups of species that are barcoded.

## Supporting Information

S1 Fig The locations for which COI Barcode Data were available from BOLD for Group I. The black triangles indicate localities with GPS coordinates, the red circles indicate countries for which GPS data were not available, and the blue squares indicate the study site (Aras River Research Station). Red and blue shaded areas indicate the general distribution areas for clades. a) *Coturnix coturnix* b) *Cuculus canorus* c) *Ficedula parva* d) *Oenanthe hispanica* e) *Emberiza citrinella* f) *Emberiza hortulana*.
(TIFF)

S2 Fig The locations for which COI Barcode Data were available from BOLD for Group II. The black triangles indicate localities with GPS coordinates, the red circles indicate countries for which GPS data were not available, and the blue squares indicate the study site (Aras River Research Station) a) *Lanius minor* b) *Acrocephalus palustris* c) *Saxicola rubetra* d) *Emberiza calandra*.
(PNG)

S3 Fig See Fig 3 caption. Red, a) *Alcedo atthis* b) *Coracias garrulus* c) *Oriolus oriolus* d) *Galerida cristata* e) *Remiz pendulinus* f) *Cettia cetti* g) *Phylloscopus trochilus* h) *Locustella luscinioides* i) *Sylvia nisoria* j) *Muscicapa striata* k) *Luscinia svecica* l) *Turdus merula* m) *Sylvia atricapilla* n) *Passer domesticus* o) *Passer montanus*
(DOCX)

S1 Table The migratory/resident status of each species, average number of individuals caught for each species, and sequence information. (XLSX)
