# Supplementary figures and images for: DNA Barcoding of Birds at a Migratory Hotspot in Eastern Turkey Highlights Continental Phylogeographic Relationships
Source: PLoS One. 2016 Jun 15;11(6):e0154454. doi: 10.1371/journal.pone.0154454 (PMC4909268; doi:10.1371/journal.pone.0154454)

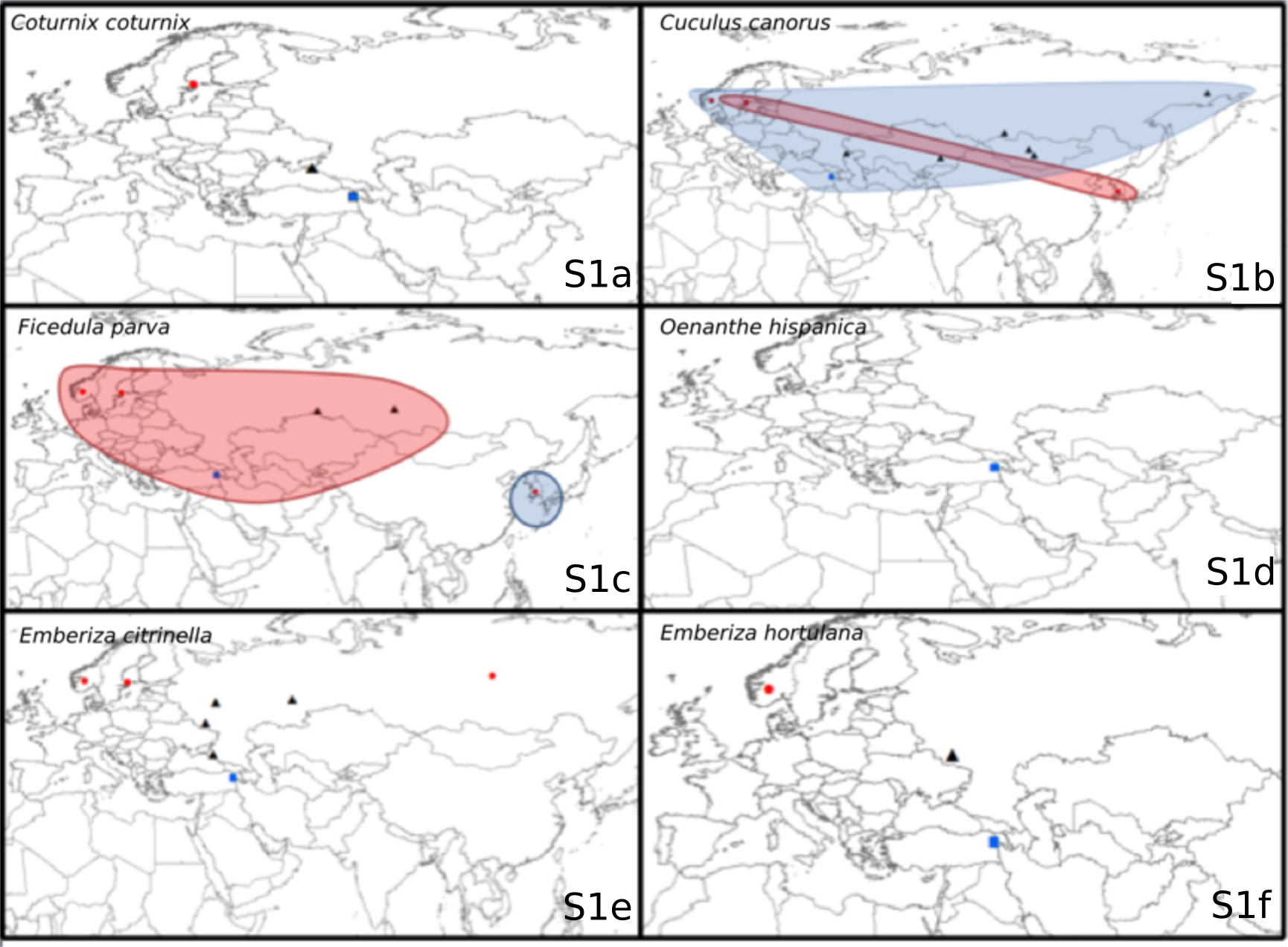

Supplement: S1 Fig — The black triangles indicate localities with GPS coordinates, the red circles indicate countries for which GPS data were not available, and the blue squares indicate the study site (Aras River Research Station). Red and blue shaded areas indicate the general distribution areas for clades. a) Coturnix coturnix b) Cuculus canorus c) Ficedula parva d) Oenanthe hispanica e) Emberiza citrinella f) Emberiza hortulana. (TIFF) [file pone.0154454.s001.tiff]

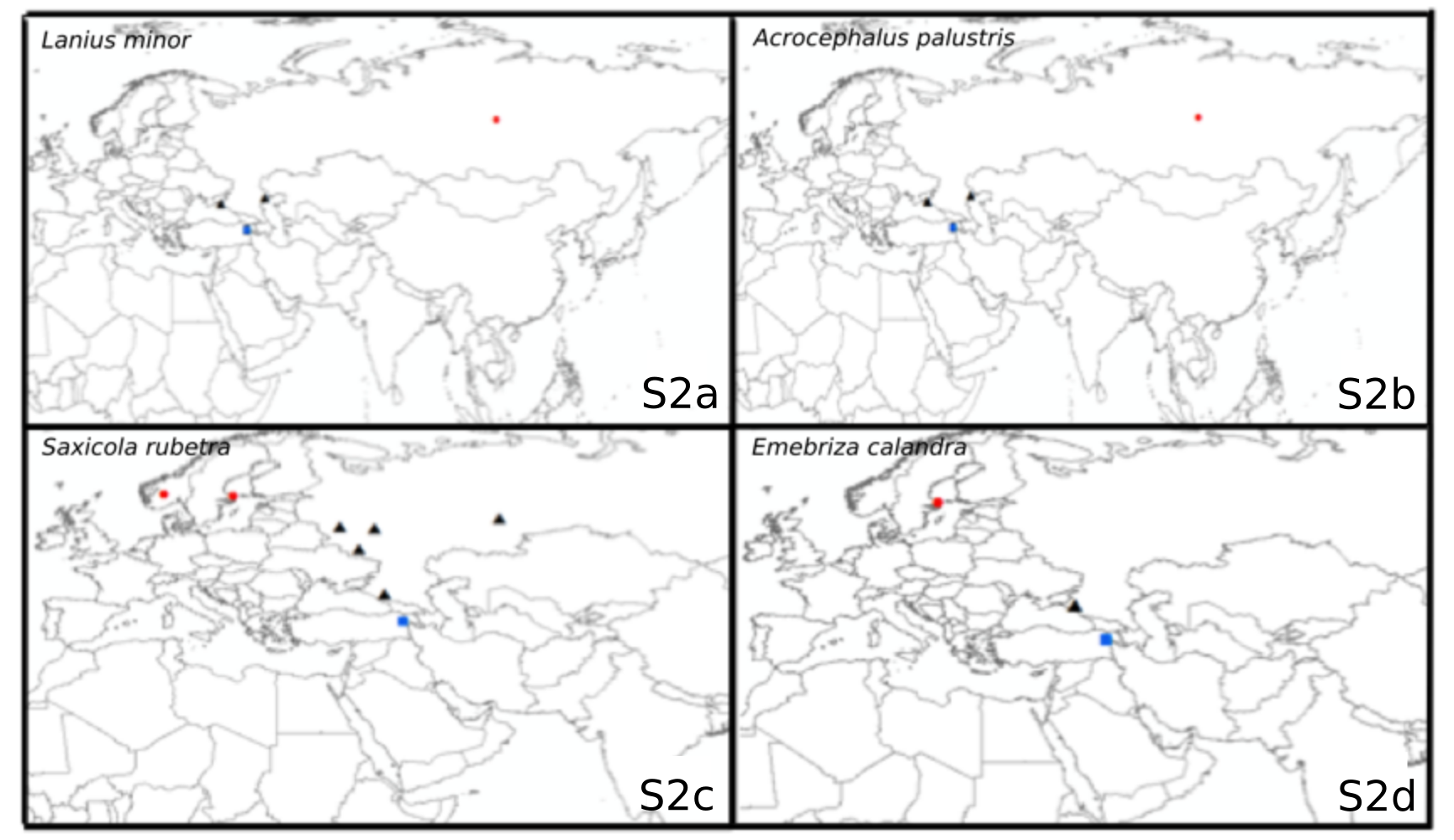

Supplement: S2 Fig — The black triangles indicate localities with GPS coordinates, the red circles indicate countries for which GPS data were not available, and the blue squares indicate the study site (Aras River Research Station) a) Lanius minor b) Acrocephalus palustris c) Saxicola rubetra d) Emberiza calandra. (PNG) [file pone.0154454.s002.png]
