# Supplementary material for: DNA Barcoding of Birds at a Migratory Hotspot in Eastern Turkey Highlights Continental Phylogeographic Relationships
Source: PLoS One. 2016 Jun 15;11(6):e0154454. doi: 10.1371/journal.pone.0154454 (PMC4909268; doi:10.1371/journal.pone.0154454)
Supplement: S3 Fig — Red, a) Alcedo atthis b) Coracias garrulus c) Oriolus oriolus d) Galerida cristata e) Remiz pendulinus f) Cettia cetti g) Phylloscopus trochilus h) Locustella luscinioides i) Sylvia nisoria j) Muscicapa striata k) Luscinia svecica l) Turdus merula m) Sylvia atricapilla n) Passer domesticus o) Passer montanus (DOCX) [file pone.0154454.s003.docx]

**S3 Fig. The locations for which COI Barcode Data were available from BOLD for *Saxicola maurus* (Group I).** The black triangles indicate localities with GPS coordinates, the red circles indicate countries for which GPS data were not available, and the blue squares indicate the study site (Aras River Research Station).. Red, a) *Alcedo atthis* b) *Coracias garrulus* c) *Oriolus oriolus* d) *Galerida cristata* e) *Remiz pendulinus* f) *Cettia cetti* g) *Phylloscopus trochilus* h) *Locustella luscinioides* i) *Sylvia nisoria* j) *Muscicapa striata* k) *Luscinia svecica* l) *Turdus merula* m) *Sylvia atricapilla* n) *Passer domesticus* o) *Passer montanus*


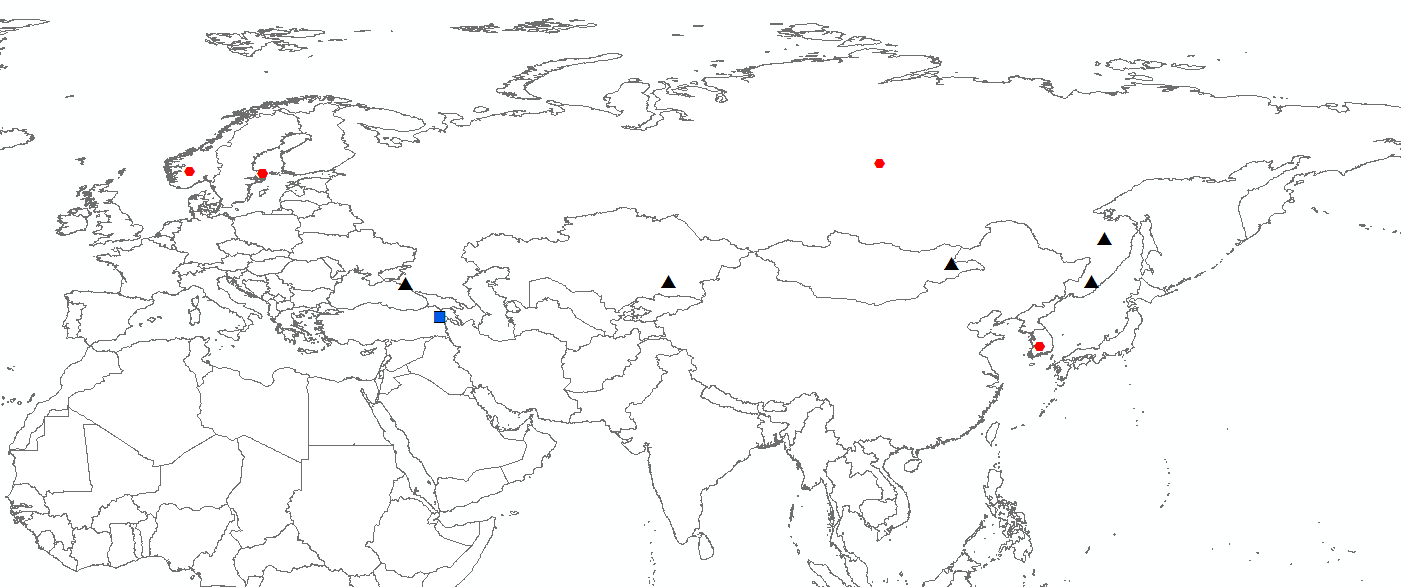


S3a


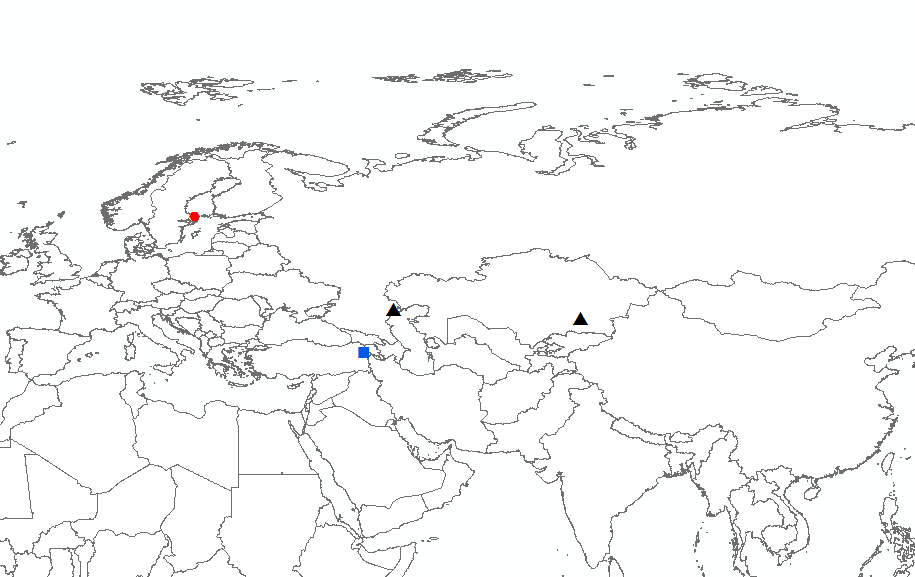


S3b


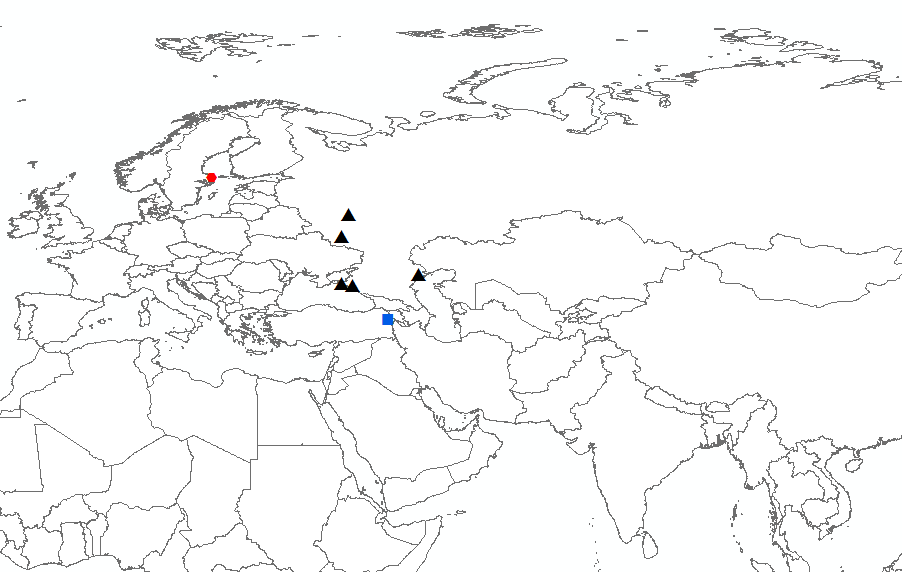


S3c


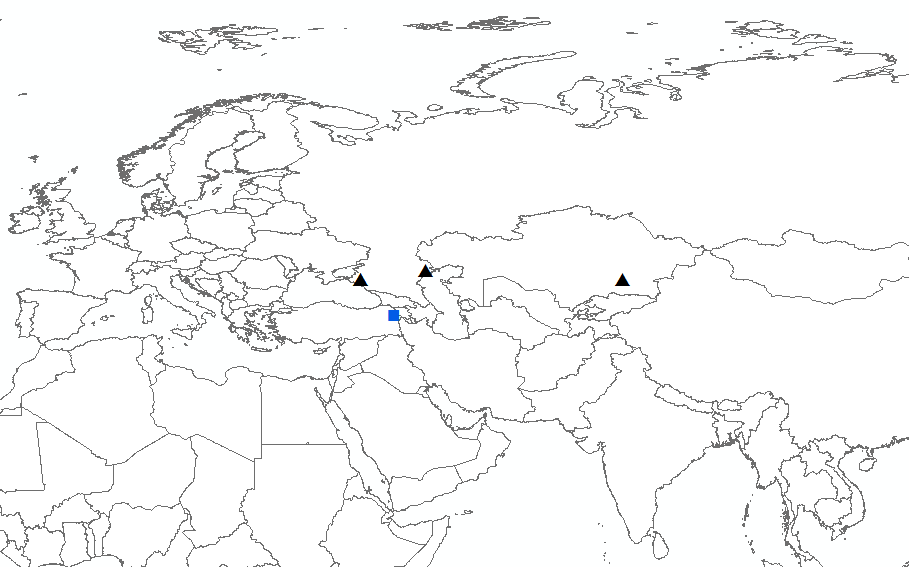


S3d


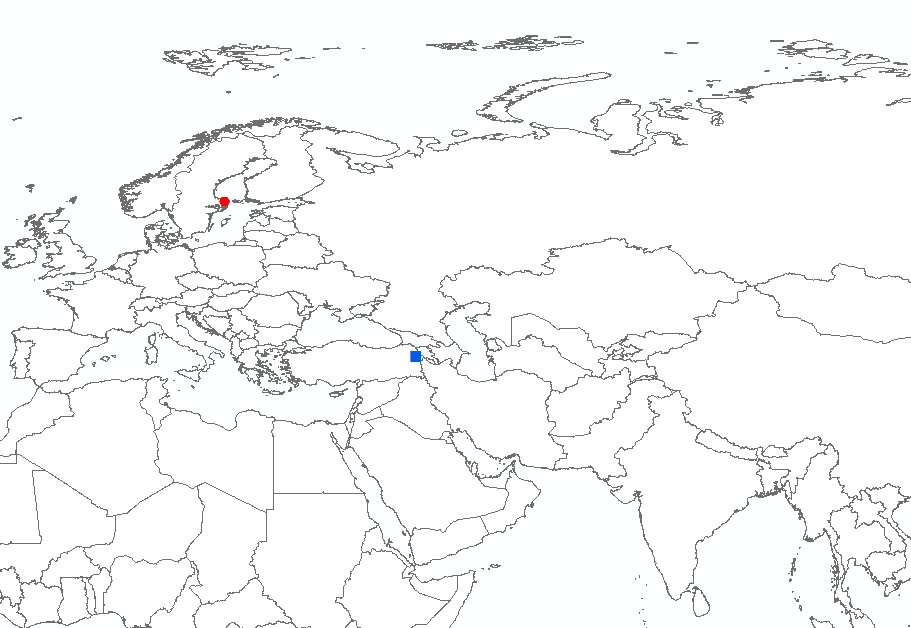


S3e


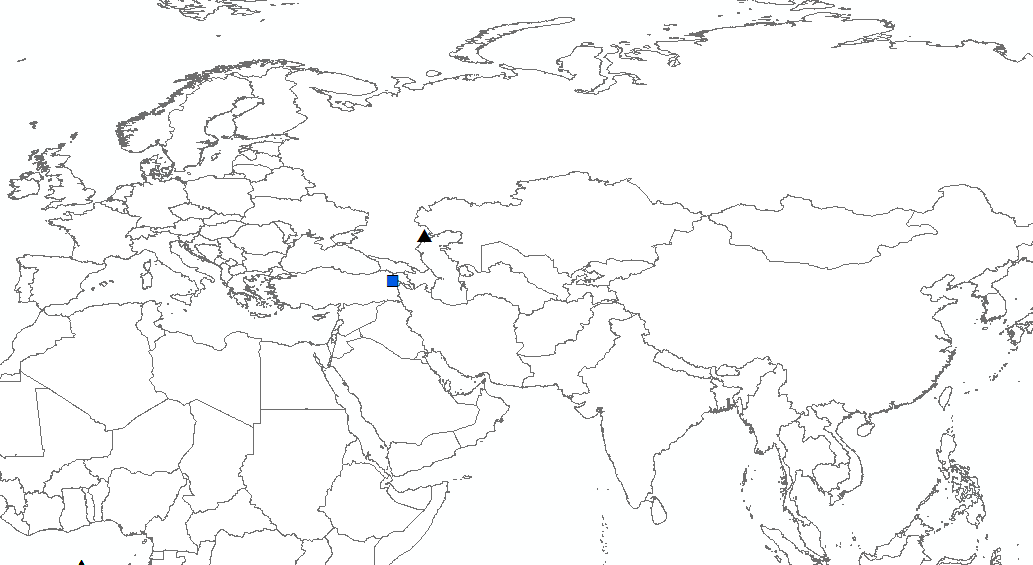


S3f


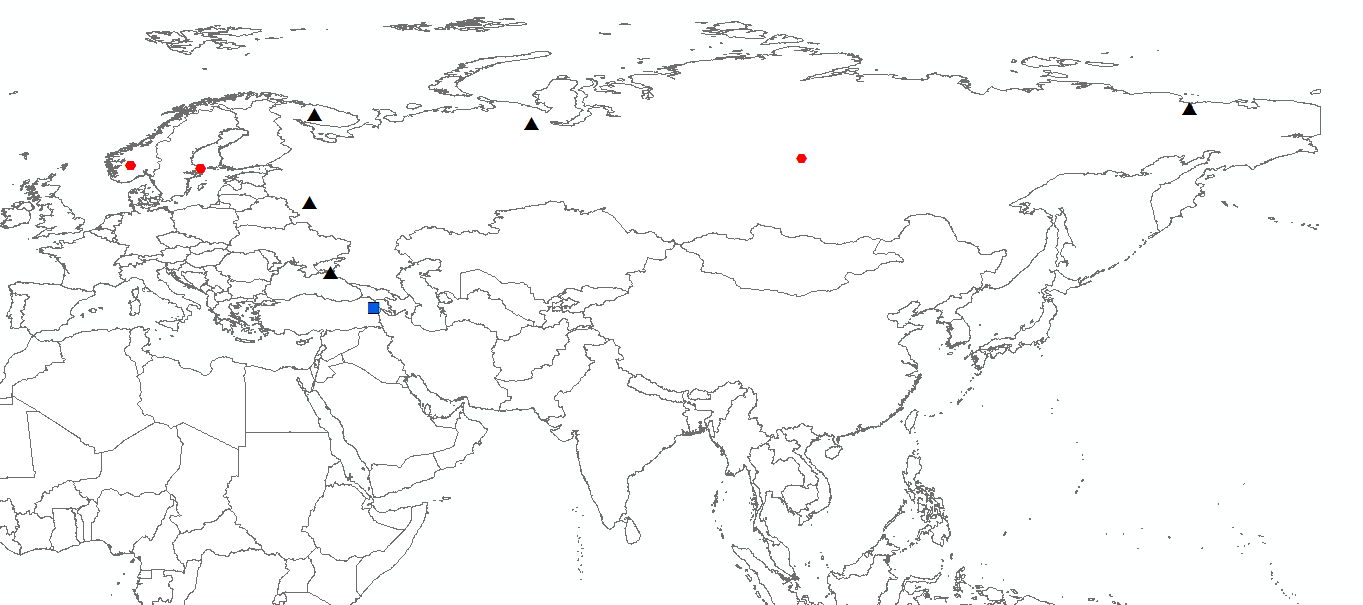


S3g


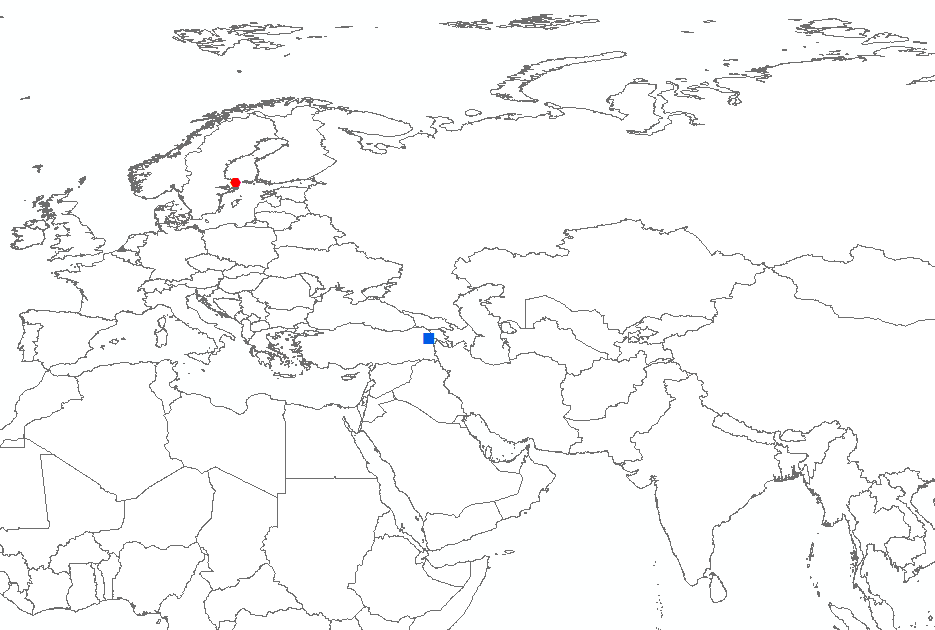


S3h


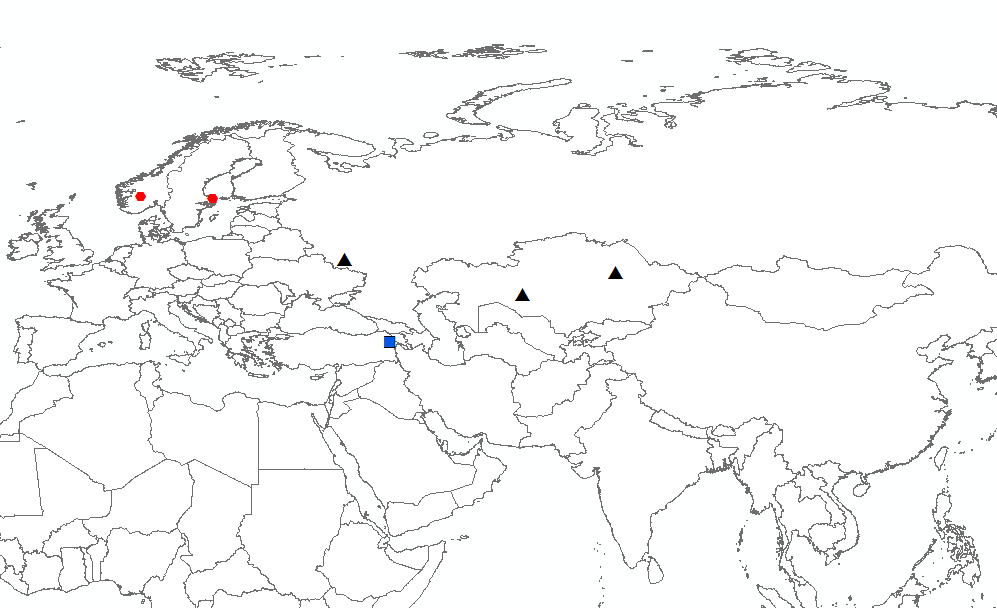


S3i


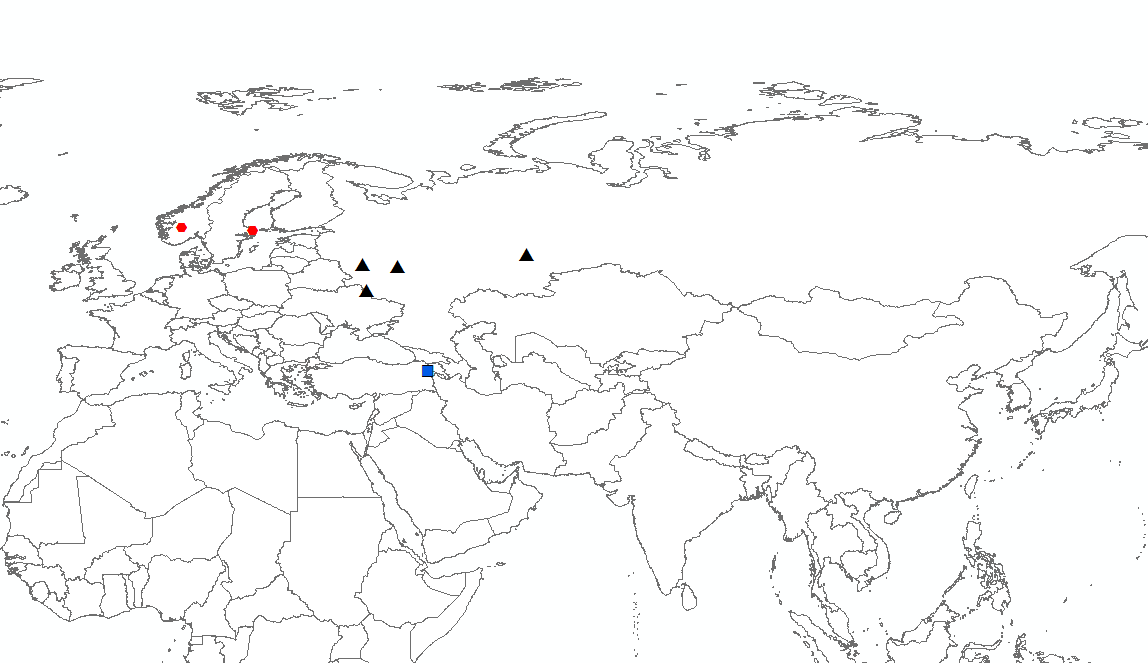


S3j


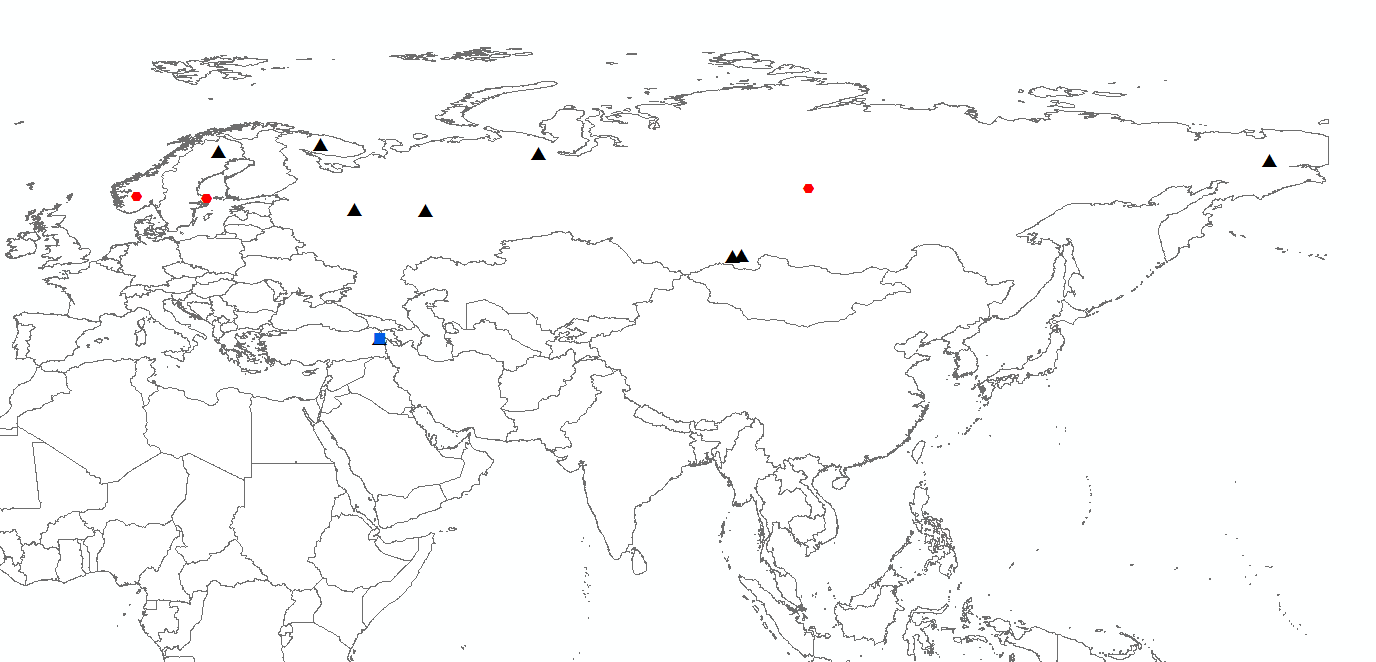


S3k


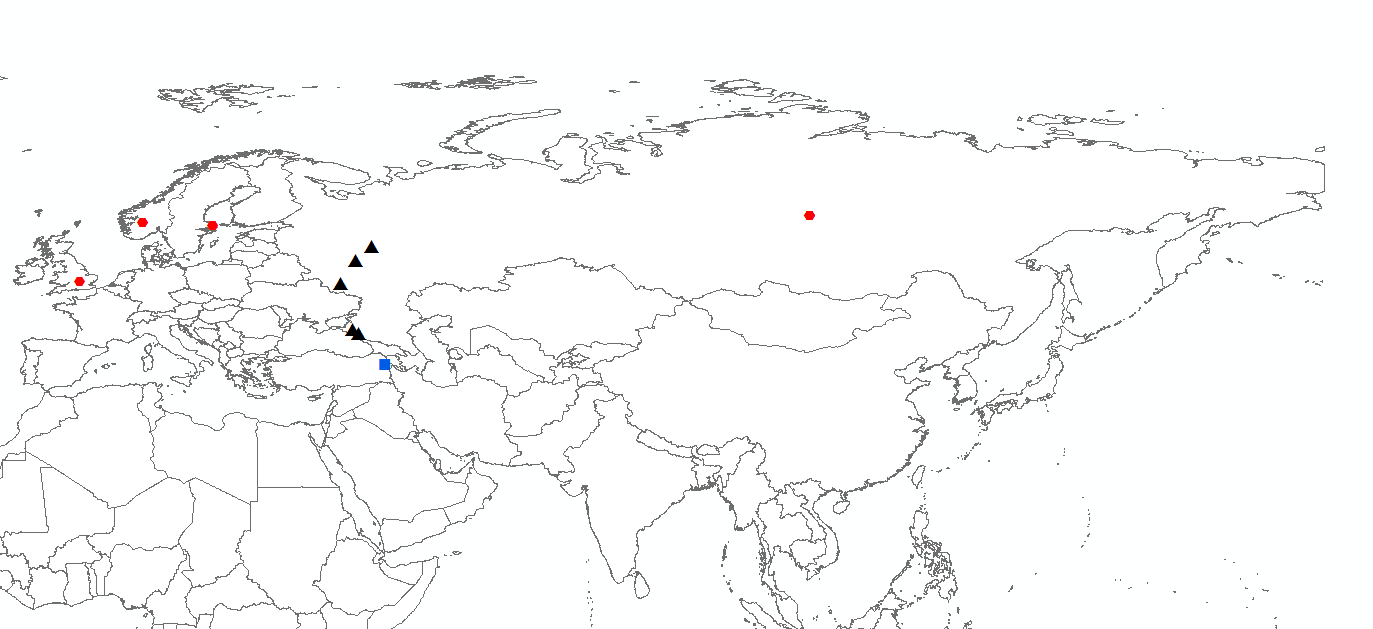


S3l


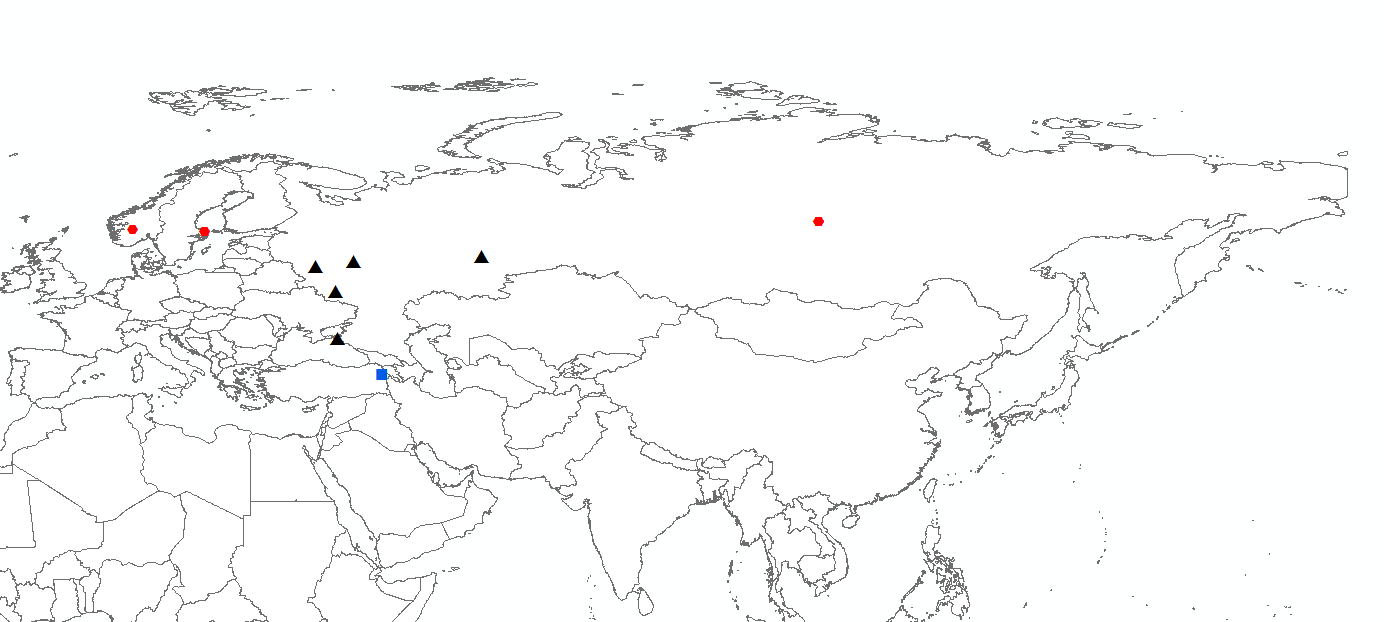


S3m


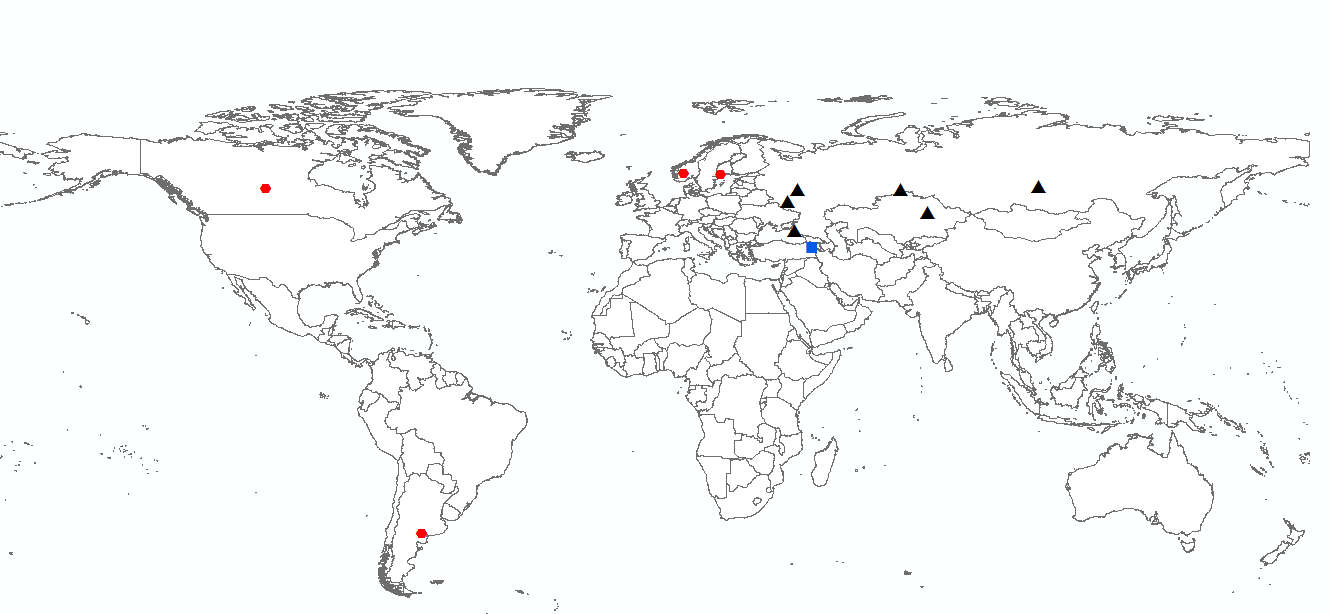


S3n


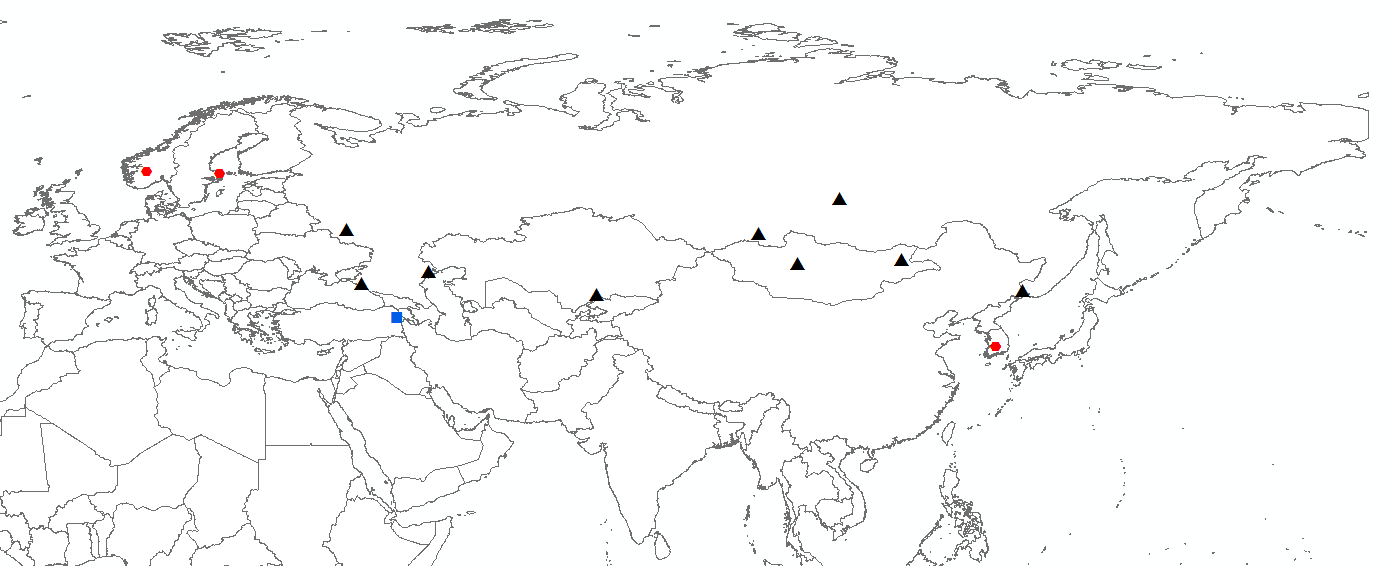


S3o
